# Population Abundance of the Endangered Galapagos Sea Lion *Zalophus wollebaeki* in the Southeastern Galapagos Archipelago

**DOI:** 10.1371/journal.pone.0168829

**Published:** 2017-01-04

**Authors:** Marjorie Riofrío-Lazo, Francisco Arreguín-Sánchez, Diego Páez-Rosas

**Affiliations:** 1 Universidad San Francisco de Quito, Galapagos Science Center, San Cristóbal Island, Galápagos, Ecuador; 2 Instituto Politécnico Nacional, Centro Interdisciplinario de Ciencias Marinas, La Paz, Baja California Sur, México; 3 Dirección Parque Nacional Galápagos, Unidad Técnica Operativa San Cristóbal, San Cristóbal Island, Galápagos, Ecuador; Department of Agriculture and Water Resources, AUSTRALIA

## Abstract

There is great concern regarding the population status of the endangered Galapagos sea lion (GSL) because it has drastically decreased over the last 30 years. We determined the population size and growth trend of the GSL in the Galapagos southeastern region (SER) at three population levels based on the available census data: 1) SER (2011–2015), including 13 rookeries on the four islands San Cristóbal (SC), Española, Floreana, and Santa Fe, comprising 58% of the archipelago’s population; 2) SC (2011–2015), including five rookeries, comprising 52% of the SER population; and 3) El Malecón (2005–2015), the largest rookery on SC and in the SER (43% of the population on SC and 22% in the SER). We also analyzed the influence of environmental variability on pup abundance in these rookeries. The current GSL population size in the SER, after applying correction factors to the counts, is estimated at approximately 2300–4100 individuals and has declined at an average annual rate (ʎ) of 8.7% over the last five years. A similar trend was determined for SC but at ʎ = 1.4% during the same period. For El Malecón, a count-based population viability analysis using a diffusion approximation approach showed that the population increased from 2005 to 2015 at ʎ = 2%. The interannual variability in pup abundance was associated with anomalies in sea surface temperature linked to oceanographic-atmospheric events, which impact the abundance and availability of prey, and ultimately may determine the population’s reproductive success. Since rookeries in the SER had different population trends, management actions should be implemented based on their specific conditions, giving priority to rookeries such as El Malecón, which, despite showing a slightly increasing population trend, still faces a high risk of extinction due to anthropogenic disturbances and environmental variability that may affect its growth and survival.

## Introduction

Currently, there is great concern regarding the population status of the Galapagos sea lion (GSL) (*Zalophus wollebaeki*), an endemic species that breeds on almost all the islands of the Galapagos Archipelago. Its overall population, estimated at approximately 16,000 individuals according to a complete census in 2001 [1], has drastically decreased (~50%) over the last 30 years [2], leading the International Union for Conservation of Nature (IUCN) to classify it as Endangered [3]. This population decrease is related to the effects of oceanographic perturbations such as the El Niño events that occur periodically in the Galapagos, leading to a lack of food resources in the marine environment [4]. Additional threats facing this species are related to anthropogenic disturbances, such as entanglement with fishing gear, marine pollution, new diseases due to human and animal contact, and habitat loss and degradation [5].

Unlike most pinnipeds in temperate zones, which aggregate on land mainly during short, highly-synchronized breeding seasons, the GSL is a non-migratory otariid that maintains small rookeries throughout the year [6]. This is because of the weak reproductive synchrony of females, which may give birth and come into estrus at any time from August to January [7,8]. The breeding season of the GSL varies slightly depending on the island. The pups’ birth season tends to begin earlier in the western region and later in the southeastern region [9], with most of the births occurring in August to October [10]. However, births may also extend into November, as has been observed on San Cristóbal Island in the eastern part of the archipelago [8,11].

For the GSL, local trophic resources are essential for breeding success and the growth of rookeries on different islands in the archipelago [11]. Since changes in the quality of foraging areas can be used as indicators of environmental degradation and of the declines in the abundance or diversity of its trophic resources, this marine mammal may be useful as an “ecosystem sentinel” in the Galapagos [8,11,12]. Because of the species’ ecological relevance, the Galapagos National Park (Dirección Parque Nacional Galapagos, DPNG) launched a management plan for the conservation of the GSL in 2012, including a standardized method of counting populations (direct counts in the rookeries during the annual census in the entire archipelago) to determine its population size and to propose appropriate management strategies for this species [13].

The GSL population in the archipelago is managed as a single unit. However, there are differences in the foraging ecology of different groups and genetic and morphological divergence between stocks from the western and central-southeastern parts of the archipelago [8,14–17], indicating that management actions at the regional level would be appropriate. Therefore, understanding the regional dynamics of this species would allow prioritized resource allocation for conservation efforts and would improve the effectiveness of management actions.

The larger rookeries of this pinniped are located in the islands of the southeastern region of the archipelago (San Cristóbal, Floreana, Española and Santa Fe) [8]. This region is dominated by the Humboldt Current, where sea surface temperatures are cool, but warmer than the western region which is characterized by high primary productivity and marine species diversity [18,19].

Five rookeries are established on San Cristóbal Island, which concentrate the major density (52%) of animals in the southeastern region of the Galapagos. El Malecón is the largest rookery on San Cristóbal Island, in the southeastern region, and in the entire archipelago [13], and it is heavily subjected to anthropogenic disturbances due to its proximity to the main town on San Cristóbal Island. Indeed, its proximity to the town has generated numerous negative interactions between the GSL and the local community [20]. The sampling effort on San Cristóbal Island, mainly at the El Malecón rookery, has therefore intensified to a monthly sampling schedule during the last 10 years. In contrast, the rookeries on Española, Floreana and Santa Fe islands have been monitored annually over the last five years.

The population growth and pup production rates in pinnipeds can vary based on many factors, including environmental variability [21–24]. Environmental perturbations such as El Niño events reduce the biological productivity levels in the marine environment, thus reducing the abundance and availability of prey. This can result in nutritional stress in the GSL, and can increase mortality rates, especially during their first years of life [2,4].

For pinnipeds, the relative population status and the overall population size and variation are best characterized by estimates of pup production [25]. However, in small sea lion populations (<1300 individuals), as found in the Galapagos, all age/sex categories can be identified and counted on land. Because a proportion of the population will be out at sea during the census, estimates of the total population size of a species usually use a correction factor with count data [26,27].

Two previous estimates of the population size of the GSL in the archipelago have been undertaken based on count data. In 1978, the population was estimated at 40,000 individuals, whereas in 2001 the population was estimated at 14,000 to 16,000 individuals. It is not clear how these estimates were derived, as these studies do not provide details regarding the correction factors used [1,6]. However, the data suggest that the population declined substantially between 1978 and 2001. There is no evidence that the population has recovered since then. On the contrary, according to Trillmich [3], assuming a pattern of exponential decline between 1978 and 2001 (with an annual decline of 3.9%), a relatively stable population from 2001 to 2014 would still represent a reduction of more than 50% of the population over the last three generations (1984–2014).

In this study, we estimated the GSL population size in the southeastern region of the archipelago using correction factors for the census data derived from the probability of observing individuals of different age categories in their rookery during the counts [28]. Census data from 2005 to 2015 were used to estimate the average annual growth rate (ʎ) and population trend of the species in the El Malecón rookery using population viability analysis, which is a quantitative method used to predict the likely future status of a population [29]. On San Cristóbal Island (including five rookeries) and in the entire southeastern region (including a total of 13 rookeries on four islands), the ʎ and the population trend from 2011 to 2015 were estimated using regression analysis. We also analyzed the relationships between pup abundance and environmental variables, and we examined the possible causes of the variability in these populations.

## Methods

### Ethics statement

This research was performed as part of the GSL population monitoring program conducted by the DPNG and the Universidad San Francisco de Quito (USFQ) under the research permits PC-30-05, PC-15-09, PC-19-12 and PC-46-15 and was approved and supported by this institution. Ethical approval was not required for this study because no animals were handled during the census.

### Study area and sample collection

**Fig 1:**
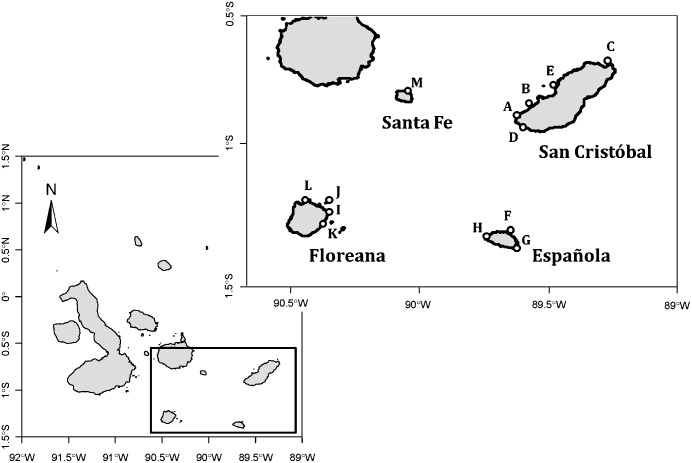
Map of the Galapagos Archipelago showing the breeding rookeries on four islands in the southeastern region censused during the study: San Cristóbal Island: A) El Malecón, B) Isla Lobos, C) Punta Pitt, D) La Lobería, E) Cerro Brujo. Española Island: F) Bahía Gardner, G) Punta Suárez, H) Punta Ceballos. Floreana Island: I) Post Office, J) Champion, K) Las Cuevas, L) Puerto. Santa Fe Island: M) Bahía Santa Fe.

The study was conducted at 13 rookeries on four islands (San Cristóbal, Española, Floreana and Santa Fe; Fig 1) in the southeastern region (SER) of the Galapagos Archipelago. Our study covers the years 2005 to 2015, with the exception of 2007, when no rookeries were surveyed. Most of the data used in this study were derived from simultaneous surveys in the rookeries: from the complete censuses on San Cristóbal Island (SC) (2011–2013); in the SER (2013); and in the whole archipelago (2014–2015). Not all rookeries were monitored with the same effort over the entire decade; however, the methodology employed in all surveys has been consistent. For example, the El Malecón and La Lobería rookeries on SC were surveyed from 2005 to 2015. All the rookeries on SC were surveyed from 2011 to 2015. In the SER, 56% of the rookeries were surveyed in 2011, 75% in 2012, 91% in 2013 and 100% in 2014 and 2015 (DPNG database).

The climate of the Galapagos Archipelago is dry with subtropical characteristics influenced by several major oceanic currents that are seasonally variable. The archipelago has two seasons: a warm season (from approximately January through May) with temperatures higher than 25°C, and a cold season (from June through December) with a temperature range between 18°C and 24°C [30]. With the exception of the 2005 and 2006 censuses, which were conducted in February, all the following surveys (from 2008 to 2015) were conducted once during October to December, corresponding to the peak of pup births in the region.

Censuses were performed on land using a direct count method [1]. Each census started at 6 am and required approximately 45 to 60 minutes to complete, depending on the size of the rookery. Two trained observers, situated in opposite boundaries of the rookery, walked along the coastline, simultaneously counting animals and identifying them by age/sex categories. The census was completed when the observers encountered each other in the middle of the rookery. Six categories were distinguished in each census: adult males, subadult males, adult females, juveniles, pups and indeterminate (unidentified animals) [1,31].

Categorized data were organized in a matrix of total counted animals per rookery and year (S1 Table). The percentage of the population that corresponded to each rookery on the island and in the SER was calculated, as was the percentage of the population counted per island and year. This calculation used only census data from years in which all of the rookeries on an island were surveyed, i.e., from 2011 to 2015 for SC and 2014 and 2015 for the other islands. The percentage of the population representing each island within the region was calculated using the census data from 2014 and 2015. The total population count of GSLs in the SER was extrapolated and estimated from the results for each year. The data from the 2001 census corresponding to each of these rookeries were used for comparisons [1].

### Population size

The GSL population size in each rookery and on each island in the SER was estimated by correcting the number of counted animals using correction factors derived from the probability of observing individuals of different age categories ashore during the counts [28]. By using the Lincoln-Petersen method, based on resights of marked animals, to estimate the population size of sea lions in the Caamaño rookery in the central archipelago over 13 years of counts, Trillmich et al. [28] calculated that the probability of observing a given adult in the rookery (*P*_ob-r_) varied between 16% (95% confidence interval (CI) = 19%-12%) for the cold/reproductive season and 23% (95% CI = 28%-18%) for the warm/non-reproductive season. For juveniles, the probabilities were 35% (95% CI = 37%-34%) during the cold season and 50% (95% CI = 52%-46%) during the warm season. According to Trillmich et al. [28], the category of juveniles in the cold season included sea lions between 1 and 4–5 years of age, but in the warm season the pups (< 1 year of age) were also included in this category. Sea lions categorized as “indeterminate” in our censuses were considered juveniles, as categorization uncertainties mostly concerned the immature category [32].

Based on these values, we assumed that the proportion of animals at sea (*p*_s_) during the counts was 1− (*P*_ob-r_/100). Thus, the numbers of adults and juveniles counted ashore (Ca) were corrected using the following equation: Ca_corrected_ = Ca + (Ca * *p*_s_). For adults, the *p*_s_ values used in the equation were 0.84 (95% CI = 0.81–0.88) in the cold season and 0.77 (95% CI = 0.72–0.82) in the warm season. For juveniles, the *p*_s_ values were 0.65 (95% CI = 0.63–0.66) in the cold season and 0.50 (95% CI = 0.48–0.54) in the warm season. The numbers of pups counted ashore during the warm season were corrected using the *p*_s_ values for juveniles in this season. When the censuses were carried out in the cold season, the sum of the corrected counts of adults and juveniles and the raw counts of pups corresponded to the population estimates in each rookery in a given year. When the censuses were carried out in the warm season, the population estimates in each rookery corresponded to the sum of the corrected counts of adults, juveniles and pups.

### Annual growth rate and population trend

A regression between years and total individuals counted between 2011 and 2015 was used to estimate the population trends in the SER and on SC. The regression between years and total pups counted was used to estimate the pup abundance trends for the same sites. The pup abundance in the SER from 2011 to 2013 was estimated from the total number of pups counted and the percentage of the censused population calculated for each year, which was 56% in 2011, 75% in 2012 and 91% in 2013. The value of the slope in the regression analysis corresponded to the average annual growth rates of the population and of pup abundance.

For El Malecón, the average annual growth rate, the population trend and the probability of extinction were determined using count data from 2005 to 2015 (without 2007) by a count-based population viability analysis (PVA) using a diffusion approximation model [33]. The PVA was only performed for El Malecón as the data from the minimum number of years required to undertake the analysis were acquired only from this rookery [34], as well as because this is the largest rookery on SC and in the SER.

The PVA estimates the general population trend and variability in population growth based on linear regressions of census data available for the rookery [33]. The probability of extinction refers to the likelihood that a rookery with a particular size and population trend reaches a critical minimum size (quasi-extinction threshold) beyond which it can no longer recover [29]. The probability of extinction requires the definition of a quasi-extinction threshold, which varies depending on populations and species [29]. In our model, we used a quasi-extinction threshold of 18 individuals, calculated from the maximum average population size recorded in El Malecón (652 individuals counted) and adjusting our estimate in proportion to that calculated for its congener in the northern hemisphere, the California sea lion (*Z*. *californianus*), for which a critical size of 50 individuals has been defined based on a maximum average population of 1811 individuals in its rookeries [35]. The PVA was performed using the program MATLAB, version R2015a (MathWorks. Inc., Natick, MA, USA).

### Pup abundance and environmental variables

The variability in pup abundance (pups counted) in each rookery of the SER throughout the years was determined using the coefficient of variation (CV). Differences in pup abundance per rookery on SC from 2011 to 2015 were tested using a two-way ANOVA, along with Tukey’s HSD test for multiple comparisons. The SC population was the only one assessed because all of the rookeries on the island were censused in those years. Census data were natural-logarithm transformed.

The effects on pup abundance of environmental variables, such as the anomalies in sea surface temperature (SST) (°C) linked to El Niño and La Niña events (measured from the El Niño 1+2 index) and the chlorophyll-a concentration (mg*m^-3^), a proxy of primary productivity, were examined in the SER, on SC and at El Malecón. The count data series were from 2011 to 2015 for SER and SC and from 2005 to 2015 for El Malecón. Chlorophyll-a values were obtained from satellite images taken at a resolution of 4.4 km in the study area (Dataset: SST, POES AVHRR, GAC, Global, Day and Night (Monthly Composite)) and are available on the ERDDAP data server from the NOAA website (http://coastwatch.pfeg.noaa.gov/erddap). The Niño 1+2 index is the three month running mean SST anomalies in the region 0–10°S, 90–80°W and is commonly used to indicate the status of the equatorial Pacific coasts, including the central and eastern islands of the Galapagos. The criterion often used to classify El Niño (La Niña) episodes is five consecutive 3-month running mean SST anomalies exceeding the threshold of ± 0.5°C. The values of the El Niño 1+2 index were taken from the NOAA website (http://www.cpc.ncep.noaa.gov/data/indices/sstoi.indices).

Spearman’s rank correlation coefficient (r_s_) was used to analyze the relationship between pup abundance in the SER and the environmental variables. This test was used because the pup data across the SER did not fulfill the assumption of homogeneity of variance (Levene’s test: p = 0.012). On SC and at El Malecón, these relationships were examined using a multiple regression analysis between the natural logarithm of the total number of pups and the average value (from August to December) of each environmental variable per year. Collinearity was ruled out, as the tolerance of each independent variable in the regression equation was 0.5 in both cases. The ANOVA, the Spearman’s rank correlation and the multiple regression analyses were conducted using Statistica version 8.0 (StatSoft. Inc., Tulsa, OK, USA). Statistical significance was defined as p < 0.05.

## Results

### Abundance and population trend

**Fig 2:**
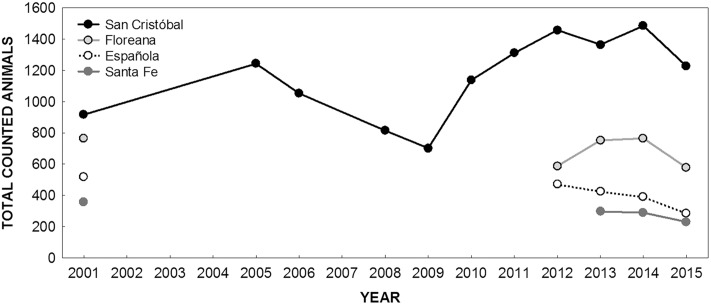
Galapagos sea lion populations counted in the southeastern islands of the archipelago. Values from 2001 were taken from [1]. Populations from 2005 to 2010 on San Cristóbal, in 2012 on Española, and in 2012–2013 on Floreana were extrapolated based on the percentage (≥ 46%) of the population censused in each year per island. In all other cases, all the rookeries of the islands were sampled. For percentages of the population censused in each rookery, see Table 1.

The estimated GSL population size in rookeries of the SER from 2005 to 2015 is shown in Table 1. The count in the SER (S1 Table) in 2015 was 2319 individuals, representing an estimated 4147 (95% confidence interval (CI) = 4099–4197) animals in total. The largest population was estimated in 2014 (5292; 95% CI = 5227–5363 individuals), based on a count of 2932 sea lions. The total numbers of counted animals per island in the SER are shown in Fig 2. Overall, there was a decrease in the population from 2014 to 2015 on all islands. The Floreana population increased from 2012 to 2014. The SC population showed fluctuations over time that might suggest different periods of growth, characteristic of the dynamics of this species.

**Table 1:** Galapagos sea lion population sizes in breeding rookeries in the southeastern region (SER) of the archipelago. Estimated values are based on corrected census data using correction factors for different age categories. The 95% confidence interval of each estimate is shown in parentheses. The average percentage of each island’s population with respect to the SER is shown next to the name of the island. The average percentage of the population represented by each rookery relative to the island is shown in the last column. Both percentage values were used to extrapolate the total population size per island and in the SER in those years during which there were missing data and the censused population was ≥ 46%.

Island/ Rookery	2005	2006	2008	2009	2010	2011	2012	2013	2014	2015	% Rookery on island
**San Cristóbal Island** 51.8%											
El Malecón	689	653	558	565	959	901	1203	1178	1126	920	42.9
	(675–708)	(638–671)	(551–564)	(558–570)	(946–973)	(891–913)	(1189–1218)	(1165–1193)	(1111–1143)	(910–930)	
Punta Pitt	–	–	–	–	625	738	691	609	908	937	31.6
					(616–635)	(730–747)	(684–699)	(602–618)	(897–919)	(926–949)	
Isla Lobos	371	292	–	–	–	490	252	431	294	199	13.4
	(373–382)	(287–300)				(486–493)	(251–254)	(428–434)	(291–297)	(198–201)	
La Lobería	218	137	172	39	72	227	173	144	159	93	6.4
	(213–224)	(134–141)	(169–174)	(38–39)	(71–73)	(225–229)	(171–175)	(142–146)	(157–161)	(92–94)	
Cerro Brujo	–	–	–	–	–	64	312	119	201	38	5.8
						(63–65)	(308–315)	(117–121)	(198–204)	(37–38)	
Sum San Cristóbal (SC)	1279	1083	729	604	1655	2420	2632	2481	2687	2188	
	(1251–1314)	(1059–1113)	(720–739)	(596–609)	(1633–1680)	(2396–2447)	(2603–2661)	(2454–2511)	(2655–2723)	(2163–2212)	
Extrapolated	2039	1727	1480	1225	2006						
Total population on SC	(1193–2096)	(1688–1775)	(1463–1499)	(1211–1235)	(1980–2035)						
**Española Island** 13.0%											
Punta Suárez						–	284	279	383	331	51.4
							(281–286)	(276–283)	(379–387)	(328–334)	
Bahía Gardner						443	418	401	208	75	32.1
						(437–449)	(412–424)	(396–407)	(205–210)	(74–76)	
Punta Ceballos						–	–	81	126	106	17.7
								(80–82)	(125–128)	(105–107)	
Sum Española (ES)						443	702	762	717	512	
						(437–449)	(694–710)	(752–771)	(709–725)	(507–517)	
Extrapolated							839				
Total population on ES							(829–849)				
**Floreana Island** 25.4%											
Post Office							446	463	384	524	39.4
							(442–449)	(457–469)	(380–389)	(518–531)	
Champion							–	313	232	144	15.4
								(309–317)	(229–234)	(143–146)	
Las Cuevas							–	–	688	261	37.6
									(677–700)	(257–266)	
Puerto							61	43	67	107	7.6
							(60–62)	(42–44)	(66–68)	(105–108)	
Sum Floreana (FL)							507	819	1371	1036	
							(502–511)	(808–830)	(1353–1391)	(1023–1051)	
Extrapolated							1080	1315			
total population on FL							(1069–1091)	(1295–1333)			
**Santa Fe Island** 9.8%											
Bahía Santa Fe								524	516	412	100.0
								(517–530)	(510–524)	(407–417)	
Sum	1279	1083	729	604	1655	2863	3840	4586	5292	4147	
Southeastern Region (SER)	(1251–1314)	(1059–1113)	(720–739)	(596–609)	(1633–1680)	(2833–2896)	(3798–3883)	(4531–4642)	(5227–5363)	(4099–4197)	
Extrapolated						5120	5152	5072			
Total population in SER						(5065–5182)	(5094–5212)	(5010–5137)			

2005–2006 = Páez-Rosas D., unpublished data; 2008–2015 = DPNG database.

The abundance patterns over time among rookeries of the SER were different. Pup abundance (Table 2) varied across years and was highly variable among rookeries of the SER (average coefficient of variation (CV) = 47.3%). The highest and lowest interannual variability in pup abundance were observed in the rookeries Cerro Brujo on SC (CV = 82.2%) and Bahía Santa Fe on Santa Fe Island (CV = 6%), respectively.

**Table 2:** Numbers of Galapagos sea lion pups counted in southeastern rookeries of the archipelago. The coefficient of variation (CV) shows the variability in the number of pups counted per rookery throughout the years. The mean CV within the southeastern region is 47.3%

Island/ Rookery	2005	2006	2008	2009	2010	2011	2012	2013	2014	2015	CV
**San Cristóbal Island**											
El Malecón	76	51	49	32	37	113	112	114	57	68	42.5
Punta Pitt					32	105	91	55	101	82	33.6
Isla Lobos	27	88				144	68	120	66	57	45.1
La Lobería	13	25	17	5	7	47	34	16	20	6	66.7
Cerro Brujo						2	8	2	7	0	82.2
**Española Island**											
Punta Suárez							59	38	68	54	19.9
Bahía Gardner						8	6	23	21	1	73.4
Punta Ceballos								3	16	7	62.7
**Floreana Island**											
Post Office							92	7	30	36	75.7
Champion								17	27	14	28.7
Las Cuevas									4	6	20.0
Puerto							10	0	9	8	58.7
**Santa Fe Island**											
Bahía Santa Fe								30	30	34	6.0

**Fig 3:**
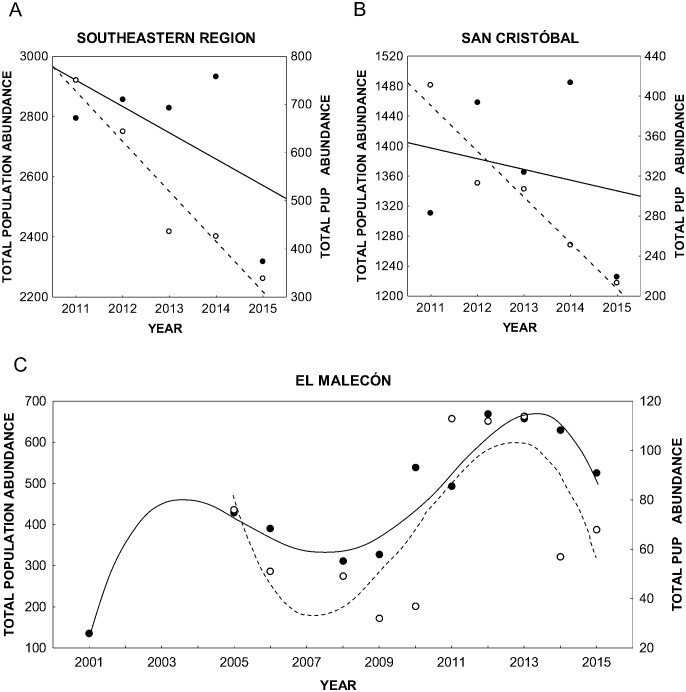
Population and pup abundance trends of Galapagos sea lions. A) Southeastern region (SER) from 2011 to 2015 (including 13 rookeries distributed on four islands); B) San Cristóbal Island (SC) from 2011 to 2015 (including five rookeries); C) El Malecón rookery from 2005 to 2015. Filled and open circles show the total population and pup population in each case, respectively. Solid and dashed lines show the population trends and the pup abundance trends, respectively. The time periods analyzed in each case correspond to those with complete data series for all rookeries, with the exception of 2011–2013 in the regional dataset, for which the total number of pups was estimated based on the total pups counted and the percentage of the population surveyed in each year. The pup abundance trends on SC and in the SER show a significant adjustment to a linear model.

The population in the SER decreased at an average annual rate of 8.7% between 2011 and 2015, (slope = -87.3, standard error (SE) = 73.4, p = 0.031, *R*^*2*^ = 0.3) (Fig 3A), while pup abundance decreased at an average rate of 10.4% during this period (slope = -103.9, SE = 16.93, p = 0.008, *R*^*2*^ = 0.9). The mean (± standard deviation, SD) number of pups was 519 ± 171. Pup abundance in 2011 was estimated at 710 individuals based on a count of 56% of the total population, whereas in 2012 and 2013, 644 and 437 pups were estimated from counts of 75% and 90% of the total population, respectively.

The SC population decreased at an average rate of 1.4% between 2011 and 2015 (slope = -14.3, SE = 37.93, p = 0.731, *R*^*2*^ = 0.04) (Fig 3B), whereas pup abundance decreased at an average annual rate of 4.6% (slope = -45.6, SE = 7.14, p = 0.007, *R*^*2*^ = 0.9). During this period, the average number of pups was 299 ± 75. There were no significant differences between years in the numbers of pups counted from 2011 to 2015 (Two-way ANOVA: F_(1,4)_ = 2.575, P = 0.07), but differences were observed between rookeries (Two-way ANOVA: F_(1,4)_ = 40.95, P < 0.05). Tukey’s HSD test for multiple comparisons revealed that the numbers of pups counted at La Lobería and Cerro Brujo differed significantly from the numbers at the other rookeries (p < 0.05).

According to the count-based PVA, the El Malecón population increased at an average rate of 2% from 2005 to 2015 (ʎ = 1.02; 90% confidence interval (CI) = 0.89–1.17) (Fig 3C). If this trend holds, there is a 37% probability of extinction of this population within 100 years and a 50% probability that the population will disappear in 125 years. The PVA based solely on pup numbers showed a decrease in the pup abundance at an average rate of 2% (ʎ = 0.98; 90% CI = 0.73–1.33). During this period, 70.9 ± 31.8 pups were counted per year.

### Pup abundance and environmental variation

There was a significant negative relationship between pup abundance and the Niño 1+2 index in the SER (2011–2015), but no such relationship was found between pup abundance and the chlorophyll-a concentration (Table 3). The multiple regression model was sufficient to explain the relationship between environmental variables and pup abundance on SC (2011–2015) (F_(2,2)_ = 12.72, p < 0.05, R^2^ = 0.92). On SC, pup abundance showed a significant negative relationship with the Niño 1+2 index but no relationship with chlorophyll-a concentration (Table 4). At El Malecón (2005–2015), the multiple regression model was weak to explain the relationship between environmental variables and pup abundance (F_(2,7)_ = 0.92, p < 0.05, R^2^ = 0.21). In this rookery, pup abundance showed no relationship with either the Niño 1+2 index or the chlorophyll-a concentration (Table 5).

**Table 3:** Spearman’s rank correlation coefficient results. Relationships between pup abundance in the southeastern region and environmental variables from 2011 to 2015; significant values are shown in bold.

Southeastern region	Variables	Spearman rank correlation coefficient results
Pup abundance vs.	Niño 1+2 index	r_s_ = -0.90, p < **0.05**
	Chlorophyll-a	r_s_ = 0.10, p > 0.05

**Table 4:** Multiple regression analysis results. Relationships between pup abundance on San Cristóbal Island and environmental variables from 2011 to 2015; significant values are shown in bold.

San Cristóbal Island	Variables	Multiple regression analysis results
Pup abundance vs.	Niño 1+2 index	t_(2)_ = -4.93, p = **0.04**
	Chlorophyll-a	t_(2)_ = -1.94, p = 0.19

**Table 5:** Multiple regression analysis results. Relationships between pup abundance in the El Malecón rookery and environmental variables from 2005 to 2015; significant values are shown in bold.

El Malecón rookery	Variables	Multiple regression analysis results
Pup abundance vs.	Niño 1+2 index	t_(7)_ = 0.29, p = 0.78
	Chlorophyll-a	t_(7)_ = 1.29, p = 0.23

**Fig 4:**
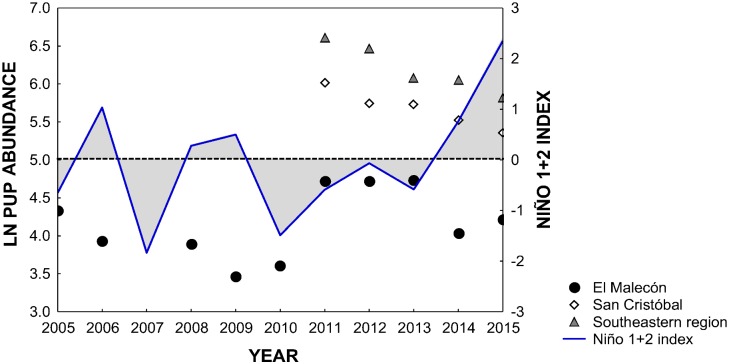
Pup abundance of Galapagos sea lions for El Malecón, San Cristóbal and the entire southeastern region of the archipelago per year in relation to the Niño 1+2 index. The Niño 1+2 index is the three-month running mean of the sea surface temperature (SST) anomalies in the Niño 1+2 region (0–10°S, 90–80°W). The values of the Niño 1+2 index plotted for each year correspond to the average of five months of data (from August to December) and were taken from the NOAA website. The Niño 1+2 index indicates the intensity of El Niño (positive values) and La Niña (negative values) events, which can be considered weak (SST anomaly of 0.5 to 0.9), moderate (1.0 to 1.4) or strong (≥1.5).

Overall, there was a negative relationship between pup abundance and the Niño 1+2 index at SC and the SER, and there were decreases in pup abundance of 10.9% on SC and 12% in the SER (Fig 4). The interannual variation in pup abundance at El Malecón rookery followed a trend similar to that described for SC and the SER, with an increasing abundance during colder years and a decreasing abundance during warmer years, but with some exceptions. Between 2005 (weak La Niña) and 2006 (moderate El Niño), there was a 9.2% decrease in pup abundance, followed by a slight decrease of 1.1% from 2006 to 2008 (normal year). During the transition from a normal year to a strong La Niña year (2010), a decrease of 7.2% in pup abundance was recorded, followed by an increase of 31% with the reduction in the intensity of the La Niña event (from 2010 to 2011). Between 2011 and 2013 (one normal year and two weak La Niña years) there was a small increase (0.18%) in pup abundance, followed by a decrease of 10.9% from 2013 (weak La Niña) to 2015 (strong El Niño).

## Discussion

The current size of the GSL population in the SER was estimated to be between ~2300 and 4100 individuals, and has declined at an annual rate of 8.7% between 2011 and 2015. The population peaked in 2014, and at present, the population size is slightly larger (~6% greater) than estimated for 2001. The SC population, which represents 52% of the GSL population in the SER, declined at an average annual rate of 1.4% between 2011 and 2015. In both the SER and SC populations, pup abundance has decreased, which will further affect the population in the coming years due to the reduced recruitment into the breeding population.

The marine habitat characteristics and the environmental conditions, which differ in each region of the Galapagos Archipelago [36], influence the habitat use of the GSL [8,15–17] and determine the population trend of its rookeries; thus, similar population trends were expected among spatially close rookeries in the same region. However, we found different abundance patterns among rookeries in the SER, suggesting that the population trend of a single rookery should not be considered representative of the trend in the entire region. Rather, each rookery should be analyzed independently over time, especially considering the high degree of philopatry of GSL to its natal breeding rookeries [37,38]. For instance, at El Malecón, the population increased at an average rate of 2% between 2005 and 2015, while the pup abundance decreased at an average rate of 2% in the same period. If this population trend is maintained, the probability of extinction within 100 years was estimated at 37%. Lengthening a time series improves the precision of the results and reduces the bias of the predicted population trend [39]. Although our estimate was based on a PVA using 10 years of count data, which is the minimum number of years required to undertake the analysis, and should therefore be considered with caution, some information useful for management may be derived from these results.

For example, the PVA is a quantitative method used to predict the status of a population [29] and is considered in criterion “E” of the Red List of threatened species of the IUCN [40]. Based on the PVA results and criterion “E”, the GSL population at El Malecón may be cataloged as Vulnerable, because the probability of extinction in the wild within 100 years is estimated at more than 10% [40]. Since the criteria of the IUCN categorize the total population of a species according to its vulnerability, the results for the El Malecón population must be considered as an exploratory assessment of the population dynamics of this rookery.

Although the prognosis of the El Malecón population is better than the actual status for the total population in the Galapagos Archipelago (Endangered), the El Malecón rookery still faces a high risk of extinction due to the reduced recruitment into the breeding population. In addition, this population is exposed to anthropogenic disturbances, such as domestic animal contact, that provide the potential for transmission of infectious diseases, such as canine distemper virus [1,3], as well as the influence of sewage and other pollution sources that may lead to habitat degradation [18]. The negative consequences of anthropogenic impacts may be amplified by synergistic interactions with the environmental variability [2], increasing the mortality of sea lions [4] and thereby affecting the survival of this rookery.

Differences in abundance among rookeries in the SER could be related to a number of factors, including mobility of animals between rookeries in the same region of the Galapagos Archipelago. A high degree of mobility has been reported between sea lions from rookeries in the central region [14,41] and between sea lions from rookeries on SC in the SER [42]. For example, adult animals of both sexes previously tagged on El Malecón have been recorded on Punta Pitt, La Lobería and Isla Lobos [42]. It is possible that these animals have used a different rookery from their home rookery as a haul-out location during their foraging sojourns [41,43]. However, it is also possible that the El Malecón population is expanding to other rookeries, which would indicate that the El Malecón population might become a source population for other rookeries or individuals from El Malecón might disperse to colonize new sites. Further study is necessary to test this hypothesis.

Different pup abundance patterns were recorded among well-established breeding rookeries on SC over the last five years. For example, La Lobería and Cerro Brujo maintained lower mean numbers of pups counted per year (25 individuals at La Lobería, and four individuals at Cerro Brujo) relative to the rest of the rookeries (90 individuals). This indicates that Cerro Brujo is less important as a breeding site for the sea lion than are other rookeries and rather this site could be used as a haul-out area for sea lions during their foraging trips. Local feeding resources are essential for breeding success and positive growth of rookeries around the archipelago [11]. The energy cost required for females to make long foraging trips and deep dives affects the reproductive success of the rookery [44,45]. Females from the same rookery within a region of the archipelago display different foraging strategies [8,41,46], which seem to be related to their needs at different stages of pup rearing [47]. It is not known whether this behavior is maintained between individuals of all rookeries in the SER. However, this may cause a differential energy cost between females of different rookeries that might be reflected in the annual pup abundance.

Climate variability strongly influences marine ecosystems, with repercussions at multiple scales, ranging from those that affect individuals to those that affect the trophic web [20]. The interannual variability in pup abundance on SC and in the SER was strongly associated with anomalies in SST. There was a decrease in pup abundance with the increase in SST anomalies from 2011 (weak La Niña) to 2015 (strong El Niño). The SST is highly correlated with other variables, such as air temperature and the concentration of chlorophyll-a in the environment [20], and it is a good indicator of the environmental variability that affects the demographics of GSLs and the abundance and distribution of their main prey species.

The primary productivity of the ecosystem can influence the abundance of sea lion prey via an upward trophic cascade [48,49] and can thus be ultimately reflected in the reproductive success of GSLs. However, we did not observe a relationship between the pup abundance and primary productivity (using the concentration of chlorophyll-a as a proxy). The reasons for the absence of such a relationship are unclear. However, our findings indicate that the El Niño index is better than the concentration of chlorophyll-a as an indicator of the effects of environmental variation on GSL abundance.

Many pinniped populations exhibit seasonal fluctuations in abundance according to the availability of their main prey species (e.g., [50,51]). However, the GSL has developed a seasonal flexibility in its diet, feeding on a variety of species but specializing in certain groups of prey species such as the whitespotted sand bass (*Paralabrax albomaculatus*), the bigeye scad (*Selar crumenophtalmus*), the Galapagos thread herring (*Ophistonema berlangai*) and the anchovy (Anchoa sp.) that alternate in frequency and abundance between the seasons [8,46]. This flexibility in the foraging behavior of the GSL has enabled it to adapt to the changing conditions of the Galapagos ecosystem and may thereby improve its survival [15].

GSLs repeatedly utilize the same feeding grounds [4] and feed on the same main prey species over time [8], even during El Niño and La Niña years [4]. The incorporation of ecologically equivalent prey species to the diet of GSL during anomalous years has been suggested as a strategy for overcoming times of nutritional stress [4]. Nevertheless, during strong El Niño events such as those of 1982–83 and 1997–98, the food availability for sea lions decreased markedly, resulting in the substantial decline of the population (by about one-third) [7]. The average annual values of the El Niño 1+2 index during these events were 2.48 and 2.54, respectively. In 2015, the average annual value of the El Niño 1+2 index was 1.67, and the SER population decreased by 22% from 2014 (previous El Niño event) to 2015 (during the strong El Niño event). If the population is reduced proportionally to the intensity of the El Niño event, a reduction of ~40% of the population is expected in 2016. There is no evidence that the population has recovered from the impacts of the two-previous strong El Niño events, and this could be related to the frequency (every 4 to 5 years) of different intensity El Niño events occurring in the archipelago. Therefore, the increased frequency of strong El Niño events could seriously limit the population recovery. This study shows the vulnerability of this species to the natural climatic variation of the ecosystem and highlights the importance of continuing studies of population dynamics and long-term monitoring the GSL population in the entire archipelago.

### Correction factors for count data

Some assumptions and caveats related to the correction factors used in this study must be addressed. The average probabilities of observing individuals ashore during the counts, on which we based our correction factors for estimating the GSL population size in the SER, were calculated by Trillmich et al. [28] using raw counts of tagged sea lions that were resighted and estimates of the population size at the Caamaño rookery while employing the Lincoln-Petersen capture-recapture estimator, which assumes a closed population. As explained by Trillmich et al. [28], for juveniles, the Lincoln-Petersen estimates were close to the number of animals tagged. However, for adults, which haul out at places other than their home rookery during their foraging trips [41,43], there may be over- or underestimates of the number of individuals who belong in the rookery. However, the fact that tagged animals in Caamaño have rarely been seen in other rookeries suggests that the assumptions of the method employed by these authors were largely met. The calculated probabilities were obtained based on 13 years of continuous surveys conducted during both the warm and cold seasons, and average probabilities and their 95% confidence intervals were calculated for both periods. We therefore are confident that the probabilities used in this study represented the proportion of animals ashore at the time of the survey relatively accurately. Since the number of animals in the rookery changes seasonally (related to the reproductive seasonality of this species), using specific correction factors for each season reduces the bias in the estimates of the population size. Finally, it is important to highlight that these corrections apply to censuses conducted directly in the rookeries and do not consider the probability of observation error in counts made at a distance (i.e. from a boat at a certain distance from the coastline).

## Conclusions and Recommendations

While the GSL population declined in the SER between 2011 and 2015, the analysis of a longer series of count data from 2005 to 2015 for the largest rookery (El Malecón) in this region and in the archipelago as a whole shows an increasing trend. This trend represents changes in the population over the last decade, so environmental variation in this time period should be considered. The El Niño 1+2 index appeared to be a good indicator of the environmental variation that affects the reproductive success of GSLs as the interannual pup abundance was associated with anomalies in SST.

The results obtained regarding the population dynamics of the species may be considered within management schemes for these populations. Since different abundance patterns were described among rookeries within the SER, adequately protecting the populations likely requires that each rookery be managed according to its specific conditions. For example, rookeries highly exposed to anthropogenic disturbances should have conservation priority and should be monitored monthly to prevent any potential new threat due to human and animal contact. In addition, those rookeries identified as important breeding sites should receive additional protection because they may become source populations for other rookeries or individuals from these important breeding rookeries may disperse to colonize new sites. The El Malecón rookery is an example of a case meeting these criteria, highlighting the priority of conserving this site.

Several key strategies for GSL conservation have recently been developed and are listed in the management plan for this species [13]. These strategies include management to reduce the contact of humans and domestic dogs with sea lions, health studies in rookeries exposed and not exposed to human disturbances [52,53], the establishment of annual population monitoring in the entire archipelago, and the standardization of count methods. However, strategies for improving the quality of the marine and terrestrial habitats of sea lions have not been fully implemented. Further studies are necessary to address some uncertainties regarding the population trends of all the rookeries, as well as all the factors that may affect their variability. We strongly recommend that the management plan be fully executed, the funds designed for monitoring be increased and maintained over time and that the census around the islands be continued in the long-term to accurately predict the GSL population growth in the archipelago. Finally, we recommend that future surveys be conducted in all rookeries at least once a year during the seasonal peak of births and be based on include several counts a week (at least five times), maintaining the same census methodology that has been employed in the last decade, thus increasing the reliability of the population estimates.

## Supporting Information

S1 Table Total counts (2005–2015) of GSLs in rookeries in the southeastern region of the Galapagos Archipelago. (DOCX)
