# Supplementary material for: Population Abundance of the Endangered Galapagos Sea Lion Zalophus wollebaeki in the Southeastern Galapagos Archipelago
Source: PLoS One. 2017 Jan 4;12(1):e0168829. doi: 10.1371/journal.pone.0168829 (PMC5215494; doi:10.1371/journal.pone.0168829)
Supplement: S1 Table — (DOCX) [file pone.0168829.s001.docx]

**S1 Table. Total counts (2005–2015) of GSL in rookeries in the southeastern region of the Galapagos Islands**

| **Island** | **Rookery** | **2005** | **2006** | **2008** | **2009** | **2010** | **2011** | **2012** | **2013** | **2014** | **2015** | **% Rookery on island** |
| --- | --- | --- | --- | --- | --- | --- | --- | --- | --- | --- | --- | --- |
| San Cristóbal | El Malecón | 429 | 390 | 312 | 328 | 539 | 494 | 670 | 657 | 630 | 525 | 42.9 |
| (51.8%) | Punta Pitt |  |  |  |  | 345 | 403 | 380 | 335 | 499 | 522 | 31.6 |
|  | Isla Lobos | 221 | 186 |  |  |  | 256 | 133 | 227 | 156 | 105 | 13.4 |
|  | La Lobería | 128 | 83 | 93 | 21 | 40 | 122 | 93 | 79 | 87 | 52 | 6.4 |
|  | Cerro Brujo |  |  |  |  |  | 36 | 182 | 67 | 113 | 22 | 5.8 |
|  | Sum San Cristóbal (SC) | 778 | 659 | 405 | 349 | 924 | 1311 | 1458 | 1365 | 1485 | 1226 |  |
|  | Extrapolated total population on SC | 1243 | 1053 | 815 | 702 | 1138 |  |  |  |  |  |  |
| Española | Punta Suárez |  |  |  |  |  |  | 154 | 153 | 208 | 182 | 51.4 |
| (13.0%) | Bahía Gardner |  |  |  |  |  | 252 | 238 | 226 | 115 | 43 | 32.1 |
|  | Punta Ceballos |  |  |  |  |  |  |  | 46 | 70 | 61 | 17.7 |
|  | Sum Española (ES) |  |  |  |  |  | 252 | 392 | 425 | 393 | 286 |  |
|  | Extrapolated total population on ES |  |  |  |  |  |  | 470 |  |  |  |  |
| Floreana | Post Office |  |  |  |  |  |  | 243 | 266 | 215 | 293 | 39.4 |
| (25.4%) | Champion |  |  |  |  |  |  |  | 179 | 130 | 81 | 15.4 |
|  | Las Cuevas |  |  |  |  |  |  |  |  | 383 | 144 | 37.6 |
|  | Puerto |  |  |  |  |  |  | 33 | 25 | 37 | 59 | 7.6 |
|  | Sum Floreana (FL) |  |  |  |  |  |  | 276 | 470 | 765 | 577 |  |
|  | Extrapolated total population on FL |  |  |  |  |  |  | 588 | 752 |  |  |  |
| Santa Fe  (9.8%) | Bahía Santa Fe |  |  |  |  |  |  |  | 297 | 289 | 230 | 100.0 |
|  | Sum Southeastern Region (SER) | 778 | 659 | 405 | 349 | 924 | 1563 | 2126 | 2557 | 2932 | 2319 |  |
|  | Extrapolated total population in SER |  |  |  |  |  | 2794 | 2855 | 2827 |  |  |  |

The average percentage of each island population with respect to the SER is shown in parentheses. The average percentage of the population represented by each rookery relative to the island is also shown. Both percentage values were used to extrapolate the total population count per island and in the SER in those years during which there were missing data and the censused population was ≥ 46%.

2005-2006 = Páez-Rosas D., unpublished data; 2008-2015 = DPNG database.
